# Characterization of the p53 Response to Oncogene-Induced Senescence

**DOI:** 10.1371/journal.pone.0003230

**Published:** 2008-09-18

**Authors:** Lidia Ruiz, Magali Traskine, Irene Ferrer, Estrella Castro, Juan F. M. Leal, Marcelline Kaufman, Amancio Carnero

**Affiliations:** 1 Experimental Therapeutics Programme, Centro Nacional De Investigaciones Oncológicas (CNIO), Madrid, Spain; 2 Unit of Theoretical and Computational Biology, Faculté des Sciences, Université Libre de Bruxelles (U.L.B.), Bruxelles, Belgium; Ordway Research Institute, United States of America

## Abstract

**Background:**

P53 activation can trigger various outcomes, among them reversible growth arrest or cellular senescence. It is a live debate whether these outcomes are influenced by quantitative or qualitative mechanisms. Furthermore, the relative contribution of p53 to Ras-induced senescence is also matter of controversy.

**Methodology/Principal Findings:**

This study compared situations in which different signals drove senescence with increasing levels of p53 activation. The study revealed that the levels of p53 activation do not determine the outcome of the response. This is further confirmed by the clustering of transcriptional patterns into two broad groups: p53-activated or p53-inactivated, i.e., growth and cellular arrest/senescence. Furthermore, while p53-dependent transcription decreases after 24 hrs in the presence of active p53, senescence continues. Maintaining cells in the arrested state for long periods does not switch reversible arrest to cellular senescence. Together, these data suggest that a Ras-dependent, p53-independent, second signal is necessary to induce senescence. This study tested whether PPP1CA (the catalytic subunit of PP1α), recently identified as contributing to Ras-induced senescence, might be this second signal. PPP1CA is induced by Ras; its inactivation inhibits Ras-induced senescence, presumably by inhibiting pRb dephosphorylation. Finally, PPP1CA seems to strongly co-localize with pRb only during senescence.

**Conclusions:**

The levels of p53 activation do not determine the outcome of the response. Rather, p53 activity seems to act as a necessary but not sufficient condition for senescence to arise. Maintaining cells in the arrested state for long periods does not switch reversible arrest to cellular senescence. PPP1CA is induced by Ras; its inactivation inhibits Ras-induced senescence, presumably by inhibiting pRb dephosphorylation. Finally, PPP1CA seems to strongly co-localize with pRb only during senescence, suggesting that PP1α activation during senescence may be the second signal contributing to the irreversibility of the senescent phenotype.

## Introduction

Among the different methods cells have to monitor external or internal stresses, the surveillance mechanism associated with the p53 gene is central. Numerous molecular studies over the years have presented p53 as an essential controller of cellular and genome integrity [1]. p53 is a master transcription factor, functionally inactive under normal conditions due to its rapid degradation by the ubiquitin ligase MDM2. A chain of events triggered in response to cellular stress upsets this precise balance, leading to the uncoupling of MDM2-driven degradation and to the ultimate accumulation and activation of p53 [2]. p53 works mostly as a transcriptional activator, with few molecules in each cell [3]. However, p53 might also act as a repressor in some instances [4]. The p53 transcriptional program includes the activation of a number of cell cycle inhibitors and proapoptotic proteins, which results in apoptosis, reversible proliferative arrest or cellular senescence [5], [6], [7].

In principle, the various outcomes of p53 activation might be influenced by quantitative or qualitative mechanisms [8]. Some studies suggest that the level of p53 output determines whether cells will enter cell cycle arrest or apoptosis. Consistent with this view, only a subset of the genes induced by high p53 levels are induced by lower p53 levels [9]. Introduction of high p53 levels into tumor cell lines induces apoptosis, while the introduction of lower levels induces only cell cycle arrest [10]. However, other studies suggest that the outcome of p53 activation is determined by factors controlled by the tissue type or by the cell genotype.

Oncogenic *Ras* can activate p53 to promote cellular senescence, limiting the transforming potential of excessive signalling [11]–[16]. This study and others have demonstrated that conditional activation of p53 in mouse embryonic fibroblast cells (MEFs) produces reversible cell cycle arrest, whereas activation of p53 in the presence of oncogenic *Ras* leads to a permanent cell cycle arrest with features of cellular senescence [17], [18]. Although oncogenic *Ras* may increase p53 levels, it is not clear whether this increase is sufficient to explain the induction of senescence.

Two different, though not mutually exclusive, models have been proposed to explain the different biological outcomes associated with p53 activation. The quantitative model implies that p53 levels are sufficient to determine the outcome. Thus, low p53 levels induce a reversible cell cycle arrest while higher p53 levels induce senescence or apoptosis. This model is supported by studies in which p53 levels may be artificially controlled with the appropriate expression systems [9], [10]. One potential mechanism that could explain such an effect is based on differential p53 affinity for p53 response elements, such that genes required for a reversible cell cycle arrest have protein products with greater affinities than those required for senescence or apoptosis.

A qualitative model of p53 action implies that non-quantitative factors controlled by a stimulus, either the tissue origin or the cell genotype influence the outcome of p53 activation. Again, two non-mutually exclusive mechanisms might support the published data. First, certain collateral signals might directly modulate p53 activity by changing the conformation of p53 or its association with various coactivators, perhaps leading to the expression of different subsets of p53 target genes. Consistent with this possibility, ionizing radiation and UV light have been shown to induce expression of different subsets of p53-dependent target genes in the same cell type [9]. Interestingly, these two stimuli induce different p53 modifications [19]–[21], raising the possibility that the activating signal may modulate p53 activity in a qualitative manner by directing p53 to different promoters [22]. Similarly, the ability of oncogenes to promote either apoptosis or senescence is correlated with different p53 modifications.

Furthermore, oncogenic Ras induces p53 phosphorylation on serine 15 and induces senescence, whereas the E1A oncoprotein does not induce serine 15 phosphorylation and promotes apoptosis. The E1A effect is dominant, since cells coexpressing E1A and Ras do not contain p53 that has been phosphorylated on serine 15, and these cells are prone to apoptosis [23], [24]. Whether this effect leads to the expression of different p53 target genes has yet to be determined. Second, it is possible that the signal produced by p53 activation is the same in different contexts and that the outcome of p53 activation is determined by how this signal is interpreted by the cell. One may envision several mechanisms by which this might occur, but an obvious possibility involves the combined action of p53 and other transcription factors such that the action of p53 on outcome-specific targets is influenced by the presence or absence of these other factors. These other factors, in turn, would be the targets for the hypothetical collateral signal. One precedent for this involves the integration of p53 and interferon signaling on the p21 promoter, which contains both p53 and IRF-1 response elements that act to synergistically induce p21 expression during a DNA damage response [25]. How different signal transduction pathways integrate to produce new biological outcomes is an important biological problem that may also have an impact on the understanding of p53.

How does oncogenic Ras convert p53 to a senescence inducer? Although it seems likely that a component of this response results from the ability of oncogenic Ras to produce quantitative increases in p53 activity via ARF-mediated inhibition of MDM2, there is compelling evidence for collateral signals that modify the outcome of p53 activation leading to senescence [17], [26]. Following the discussion above, it is formally possible that oncogenic Ras directly modulates p53 activity or, instead, produces cellular changes that reinterpret the p53 signal.

One potential mechanism may involve the ability of Ras to induce PPP1CA (the catalytic subunit of PP1α) expression, regulating senescence in a pRb-dependent manner [18]. pRb is involved in the SAHF, maintaining long-term inhibition of E2F-dependent transcription through changes in the packaging status of chromatin [27].

To characterize the p53 response during growth arrest and senescence, this series of experiments compares p53-dependent transcription in different situations involving proliferation, reversible arrest, replicative senescence or Ras-induced senescence.

## Results

### p53 levels and phenotype

**Figure 1:**
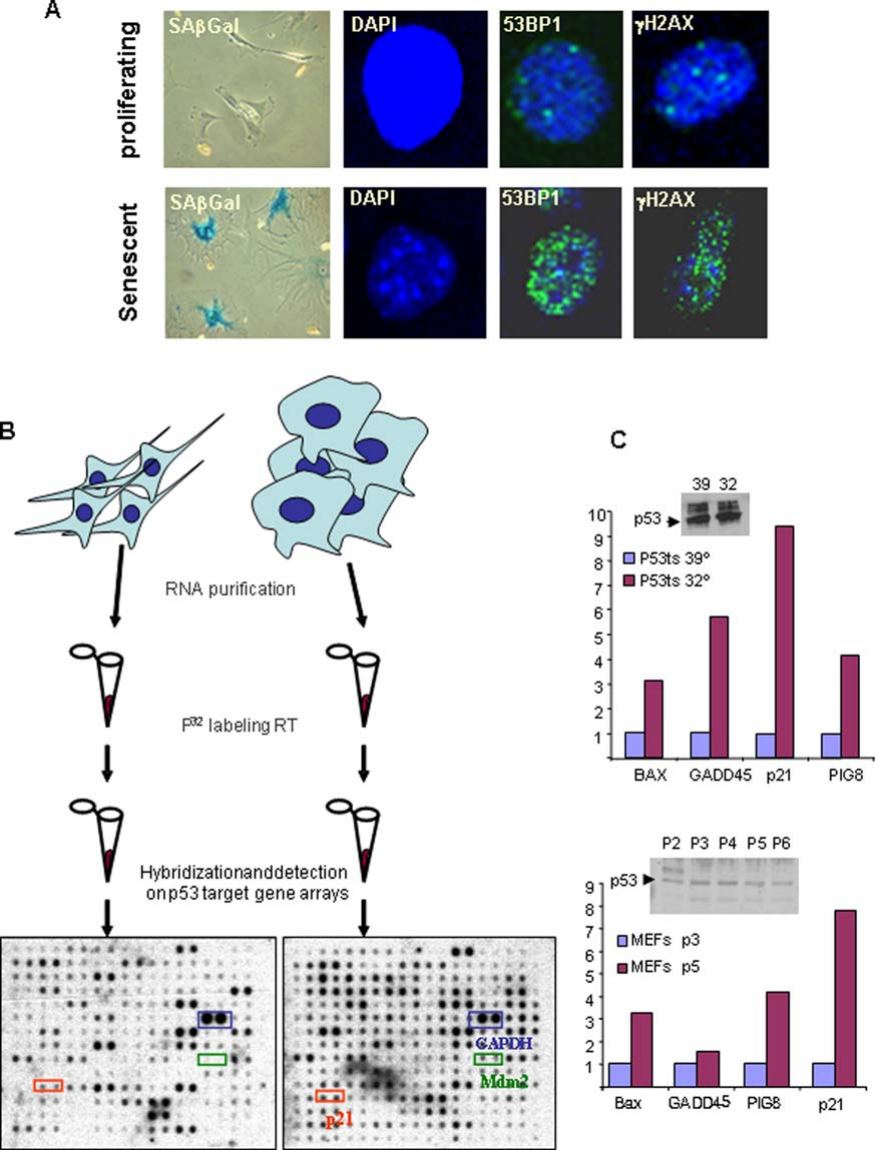
Experimental system. A) Molecular markers used to identify senescence. B) Scheme of the procedure (see M & M) and 2 representative images of the dot blot obtained after hybridization. C) Comparison of several conditions of well-known activated targets of p53. Western blot showed no variation among p53 levels under comparable conditions.

To assess if differences in p53-dependent transcription play a role in reversible arrest or senescence, this study took advantage of the mouse embryo fibroblast (MEF) cell system that allows easy manipulation of cellular stresses in otherwise homogeneous conditions. For instance, conditional activation of the p53 pathway in MEFs is known to trigger reversible cell cycle arrest, whereas activation of p53 in the presence of oncogenic Ras leads to permanent cell cycle arrest with features of replicative senescence (Figure 1A) [16].

To induce replicative senescence, wild-type and p53-null (p53 −/−) embryos were generated from crosses between heterozygous p53 knock-out mice. From wild-type embryos, MEFs were generated and grown until replicative senescence was reached (approximately at passage 5, corresponding to 10 population doublings). We extracted mRNA under these conditions, i.e., terminally arrested with senescence features (P5), and also from exponentially growing MEFs (early passage, P3). Other stress conditions leading to senescence were produced as follows. Wild-type MEFs growing at early passage were infected with retroviruses carrying oncogenic Ras (Val12-Hras). Cells were selected for retrovirus insertion and once they reached senescence (corresponding to approximately passage 3), mRNA was extracted (P3+ras). P53-null MEFs were infected with viruses carrying the 135V thermosensitive mutant of p53 that induces cellular arrest at permissive temperature (32°) [28]. These cells (p53ts), while maintained at restrictive temperature (39°C), were infected with viruses carrying oncogenic Ras (p53ts-ras), which induces senescence when shifted to permissive temperature [17]. For a summary of conditions and the resulting phenotypes see Table 1.

**Table 1:** Summary of cell lines and conditions used in this study.

Cell line	Genotype	phenotype
**p53−/−**	p53−/− MEFs	Growth at 32° and 39°
**p53−/−;ts** (p53ts)	p53−/− MEFs with p53val135	Growth at 39° reversible arrest at 32°
**p53−/−;ts-ras** (p53ts-ras)	p53−/− MEFs with p53val135	Growth at 39°; senescence at 32°
**p3**	naïve MEFs, 6 population doublings	Growth at 37°
**p5**	naïve MEFs, 10 population doublings	senescence at 37°
**P3+Ras** (Ras)	naïve MEFs+Ras-val12, 6 population doublings	senescence at 37°

The abundance of p53 did not change among different passages reaching replicative senescence, or between the restrictive or permissive status in the case of the overexpression of the thermosensitive mutant of p53 (Figure 1C). Therefore, this study first measured broad p53-dependent transcription (Figure 1B). We measured the expression of 122 p53 target genes using Dot Blot arrays in the different proliferating and arrested cellular scenarios discussed above (See Figure S1 for a list of the 122 genes analyzed). The increased transcription rates of important p53 target genes such as Bax, GADD45, p21 and PIG8 confirm the activation of p53 in both senescence systems (Figure 1C).

**Figure 2:**
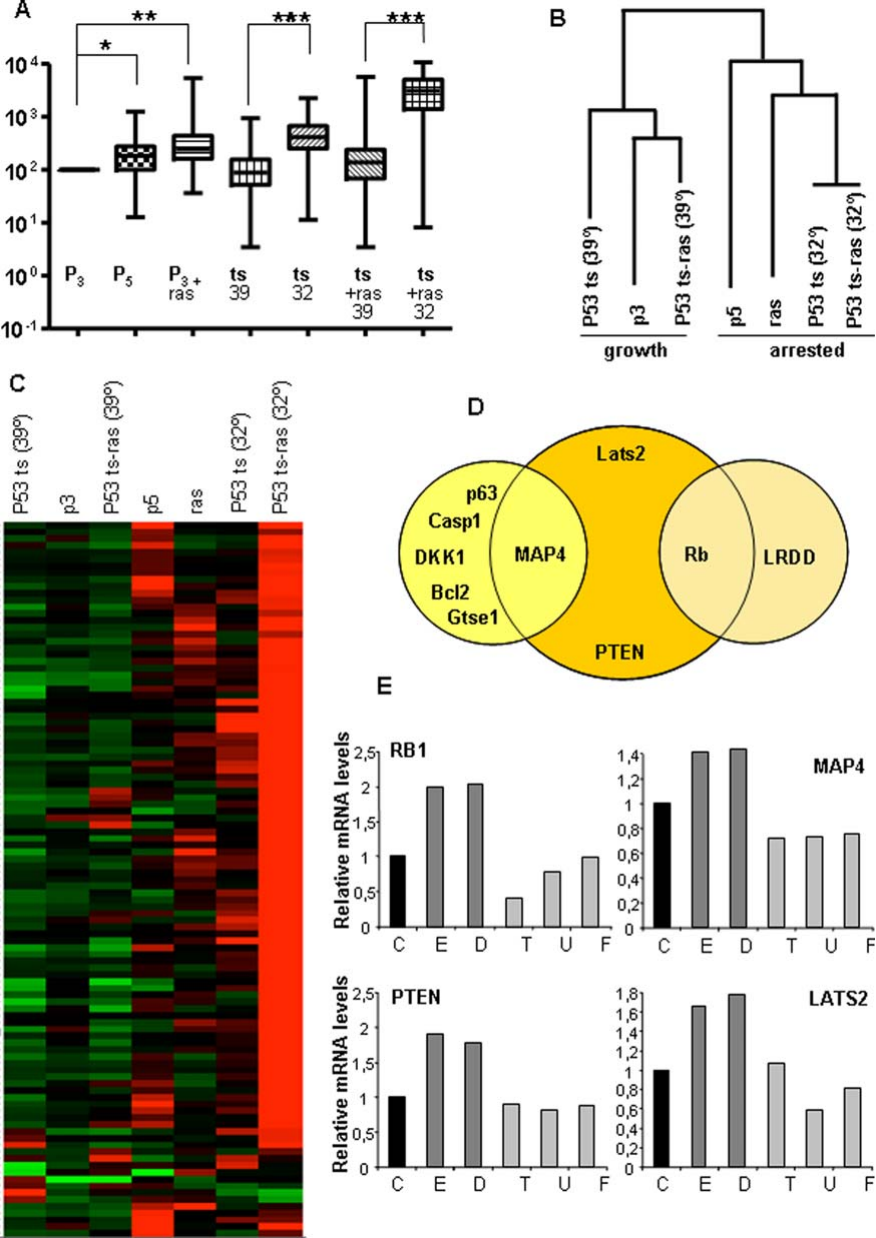
Analysis of the p53-dependent transcriptional signature. A) Comparison of the levels of 122 transcriptional targets of p53 at different conditions of p53 activity. See text. Data normalized against β-actin were compared to the proliferative condition P3 to evaluate its statistical significance. Statistical analysis was performed by paired t-test. (_*_) = p<0.05; (_**_) = p<0.005; (_***_) = p<0.001. B) and C) Analysis of the expression values of 122 transcriptional targets of p53 under different cellular conditions, which led either to proliferation or to growth arrest. Clustering analysis. Hierarchical clustering was performed using the function hcluster (package amap) of the free statistical software R. See M & M. The expression level of each gene, relative to its median expression level across all conditions, was represented by a color, with red representing expression greater than the median, green representing expression less than the median, and the color intensity representing the magnitude of the deviation from the median. D) Feature selection. Since the number of genes is much greater than the number of conditions, we used penalized regression methods. See text and M & M for more details. E) Validation of the feature selection by quantitative PCR. See text and M&M. We determined Lats2, DKK1, pRb and PTEN mRNA levels after 24 hts treatment of HCT116 cells with the indicated treatment, by quantitative PCR. Cyclophilin (ref. 4326316E), an endogenous control, was used to normalize variations in cDNA quantities from different samples. Each reaction was performed in triplicate with cDNA from normal and tumor tissue from each patient studied. C shows untreated samples. E: Etoposide, D: Doxorubicin, T:Taxol, U: UCN-01, F:Flavopiridol. Data shows average of three determinations.

We observed that the arrest of MEFs at senescence (P5) and after Ras-induced senescence (P3+ras) correlated with a net increase in p53-dependent transcription (Figure 2A). Similarly, cells arrested after p53 activation (ts and ts-Ras at 32°C) also showed, as expected, a significant increase in p53-dependent transcription (Figure 2A). Therefore, we had a genetically homogeneous system with different levels of p53 activity measured with respect to 122 p53 transcriptional targets. It was possible to ascribe a phenotype to each level of p53 activity (Table 1). There were three conditions of proliferating cells: (1) P3, (2) cells with mutant p53 at restrictive temperature (null p53), and (3) cells showing basal levels of p53 activity. There was also one condition of replicative senescence with a moderate increase of p53 activity (P5). Oncogenic Ras activation seems to induce higher levels of p53 activation with similar senescent phenotypes (P3+ras). However, elevated levels of p53 do not always induce senescence as p53ts cells at permissive temperature are reversibly arrested, but p53 activity is higher than in the two previous conditions displaying senescence. As before, oncogenic Ras expression switches the cell from arrest to senescence, also increasing the relative p53-dependent transcription (Table 1 and Figure 2A).

Therefore, arrest vs. senescence is not determined by the relative levels of p53 activity alone.

### Specificity of the senescence response

To study the expression pattern of p53-responsive genes during arrest or senescence in order to compare both processes and to ascertain what gene or genes may play a crucial role in the proliferating or arrested cell phenotypes, we performed a hierarchical clustering of the different cellular conditions on the basis of pattern similarity (see Materials and Methods). In Figures 2B and 2C we observed that the conditions are separated into two groups corresponding to the arrested (right side) and proliferating (left side) phenotypes. The cell lines are grouped together on the cluster dendogram by the activation or inactivation of p53 and not by the presence or absence of the Ras oncogene. This is clear in wild-type MEFs growing at passage 3 (P3), which have low levels of p53 activation compared to arrested wild-type MEFs in passage 5 (P5), which have p53 highly activated. However, it is interesting that the most extreme condition, p53 activation in the presence of oncogenic Ras, triggers an enhanced transcriptional response (Figures 2A and 2C, lane p53ts-Ras [32°]). See below.

Although all the physiological conditions that lead to growth-arrest onset are clustered together and all the transcripts considered are p53-dependent, it is clear that there are some genes whose enhanced activation (relative to their median expression level over all cellular conditions) is specific to each particular condition (Figure 2C and Table 2). These genes might serve as specific marker genes. However, no concurrent senescence signature could be observed, indicating that the senescence program is not determined by the specificity of the p53 response.

**Table 2:** Condition-specific genes.

P5 Replicative	Ras Oncogenic Stress	p53ts(32°) Growth arrest
IGF-R	p63	GML
MAP4	CycB1	Bak
ZAP70	Krt2-8	Bax
Wig1	Krt1-15	PTGF
PIG8	Pmaip1	Igfbp6
IL6	DKK1	Bcl6
P73	PUMA	PPM1D
Lats2	Mgmt	Tyr
Bax	Pold1	MDR1
Jun	Lats2	Thbs1
LRDD/PIDD		Kai1
Pthlh		Btg2
Waf1		MAP4
Igfbp3		Tst
MST1		RB1
RB1		IGFR
Hic1		p14-ARF

This table represents the genes that are the most representative (relative to their median level–based on all conditions) in each particular arrested condition. A threshold equal to 2.30 was chosen for the ratio. Genes are arranged from the lowest to highest ratio.

Next, applying a penalized least-squares regression technique with an L1-type penalty to the expression data (see Materials and Methods) it was possible to identify four p53 target genes among the 122 genes studied as the most relevant markers for predicting the proliferating or arrested phenotype of each cellular condition. These four relevant genes are: MAP4, PTEN, Lats2 and Rb1 (Figure 2D). Furthermore, combining L1- and L2-norm penalties allowed small subgroups of additional genes that are highly correlated with the main predictors to be extracted. This study identified five more genes closely related to MAP4 behavior: p63, caspase1, DKK1, Bcl2 and Gtse1; as well as LRDD, related to Rb1. This robust set of p53 target genes molecularly defines a minimal footprint to identify a p53-dependent arrest.

In order to confirm the p53-dependent arrest footprint defined by these markers, we measured the p53-dependent transactivation of 4 among the selected genes by qRT-PCR in HCT 116 p53+/+ cells treated with different DNA-damaging agents. p53 protein is present at low levels in resting cells but after exposure to those agents as well as to other stressing stimuli, it is stabilized and activated by a series of post-translational modifications. These modifications leave p53 free from mdm2, an E3 ubiquitin ligase that ubiquitinates it and facilitates its degradation by the proteasome [5]. p53 stabilization and activation is followed by cell-cycle arrest. To ascertain whether the transcription of this set of genes also depends on other chemotherapeutic drugs that act through p53-independent mechanisms, we also treated the cells with compounds that do not directly cause DNA breaks. Only the treatment with the topoisomerase inhibitors Etoposide and Doxorubicin induced an activation of the transcription of PTEN, Lats2, Rb1 and MAP4 (Figure 2E). However, we did not detect increase of these genes by Taxol, flavopiridol or UCN-01.

### Downregulation of p53 response without senescence

**Figure 3:**
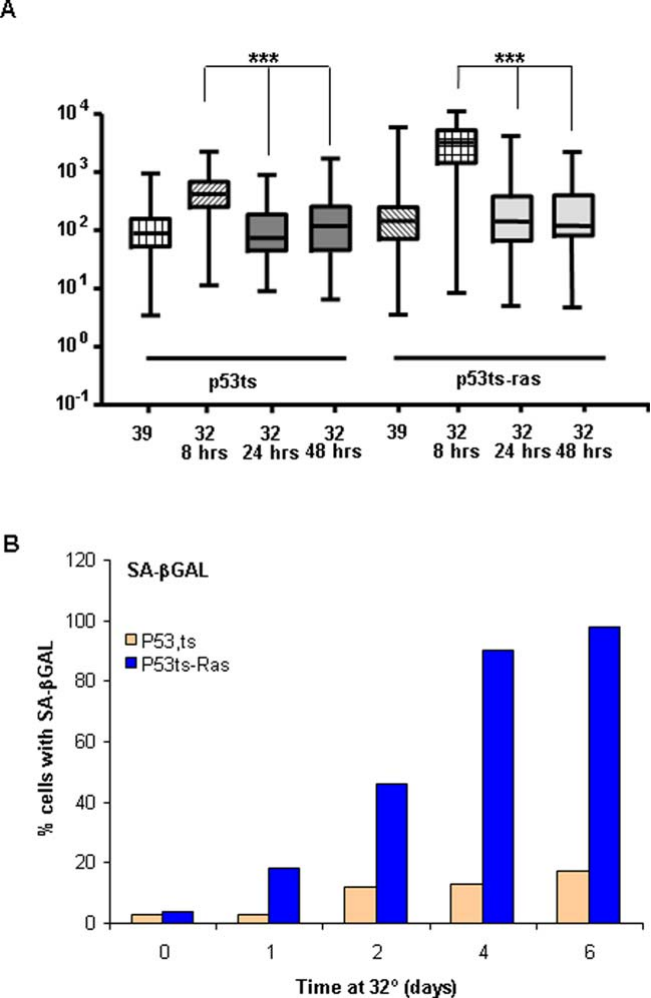
P53 activity is downregulated maintaining senescence. Cells were plated in 10-cm-diameter plates. Cells were grown at 39°C (i.e., never incubated at 32°C) or arrested for the indicated times at 32°C. Cells were harvested and RNA collected for A, or stained for SA β-GAL for B. A) Comparison of the levels of 122 transcriptional targets of p53 at different times after p53 activation. Data normalized against β-actin was compared to the proliferative conditions at 39°C to evaluate statistical significance. Statistical analysis was performed by paired t-test. (_*_) = p<0.05; (_**_) = p<0.005; (_***_) = p<0.001. B) More than 400 cells were visually analyzed for SA β-GAL staining as described in Figure 1A. Data represent the percentage of cells showing SA β-GAL staining.

P53 transcription seems to define only arrest, and not senescence, suggesting the existence of a p53-independent signal necessary to convert the reversible arrest into senescence. To explore this, we analyzed whether sustained p53 activation might induce senescence without a second signal. In the same p53-induced transcriptional setting, we analyzed the activation of p53 during long periods and its correlation with the appearance of senescence. After an initial activation, general p53-induced transcription seems to decay at 24 hrs; this downregulation is maintained for long periods even in the presence of Ras activation (Figure 3A). However, senescence features are only maintained in the p53ts-Ras cells incubated at 32°C (Figure 3B). We found that in cells carrying activated p53 only, senescence is not induced despite a long period of growth arrest (up to 6 days). These data support the finding of Ferbeyre et al. [17], that growth arrest and senescence are two independent phenotypes; the permanence of growth arrest does not induce senescence unless another signal is involved.

**Figure 4:**
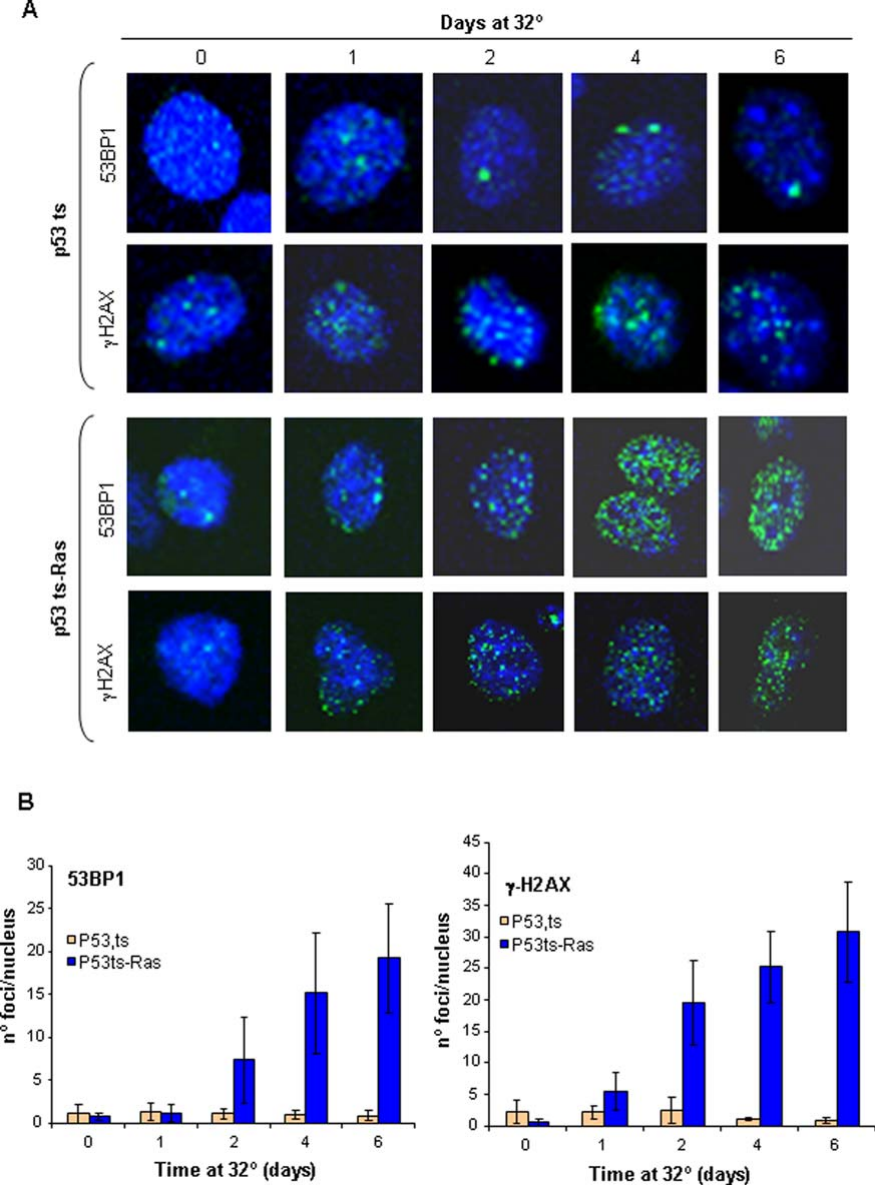
Enforced growth arrest does not induce senescence. P53ts or p53ts-Ras cells were grown at 39°C or incubated at 32°C for different times as indicated. Cells were fixed and stained with DAPI to identify the nuclei, or with antibodies against 53BP1 or phosphorylated gH2AH. A) Representative picture. B) Foci of >60 nuclei of each condition were counted and data represented as the average of the number of foci per nuclei. Bars = StDev.

Finally, to confirm that the cells in long-term arrest have not suffered molecular changes that might indicate a switch to senescence, we analyzed 53BP1 and γH2AX phosphorylation at the senescence-associated DNA foci. As before, p53ts and p53ts-Ras cells were cultured at 39°C, then were moved to 32°C and maintained for up to 6 days at restrictive temperature. Cells were taken at different time points and analyzed for the presence of DNA-damage foci labeled by 53BP1 and γH2AX phosphorylation as markers for cellular senescence (Figure 4). One or two 53BP1 and γH2AX foci appear with cell proliferation, and the same number of foci was maintained in p53ts arrested cells even after 6 days of growth arrest. However, p53ts-Ras cells showed a strong increase in the number of foci per nuclei after 48 hrs of arrest (Figure 4); this was maintained despite p53-transcriptional downregulation.

These data, which are consistent with previous observations [17], [26], indicate that initial p53 activation is required to induce growth arrest. However, a second Ras-dependent signal seems to be required to stabilize the arrest as irreversible senescence.

### PPP1CA contributes to growth arrest stabilization in senescence

The application of a retroviral-based genetic screen yielded an antisense RNA fragment against PPP1CA, the catalytic subunit of PP1α. Loss of PPP1CA function bypasses Ras/p53-induced growth arrest and senescence [18]. It was found that oncogenic Ras promotes an increase in the intracellular level of ceramides, which may increase PPP1CA activity, contributing to senescence. PP1α has been identified as the protein phosphatase responsible for the dephosphorylation of pRb [29]; this has been related to the growth arrest response [30]–[32]. When cells are actively growing, the hyperphosphorylated form of the Rb protein (ppRb) predominates. On the contrary, when cells are delayed in their growth, the hypophosphorylated form of the Rb protein (pRb) is the most abundant. Thus, enforced pRb dephosphorylation might contribute to the arrest to senescence transition [27], [33].

**Figure 5:**
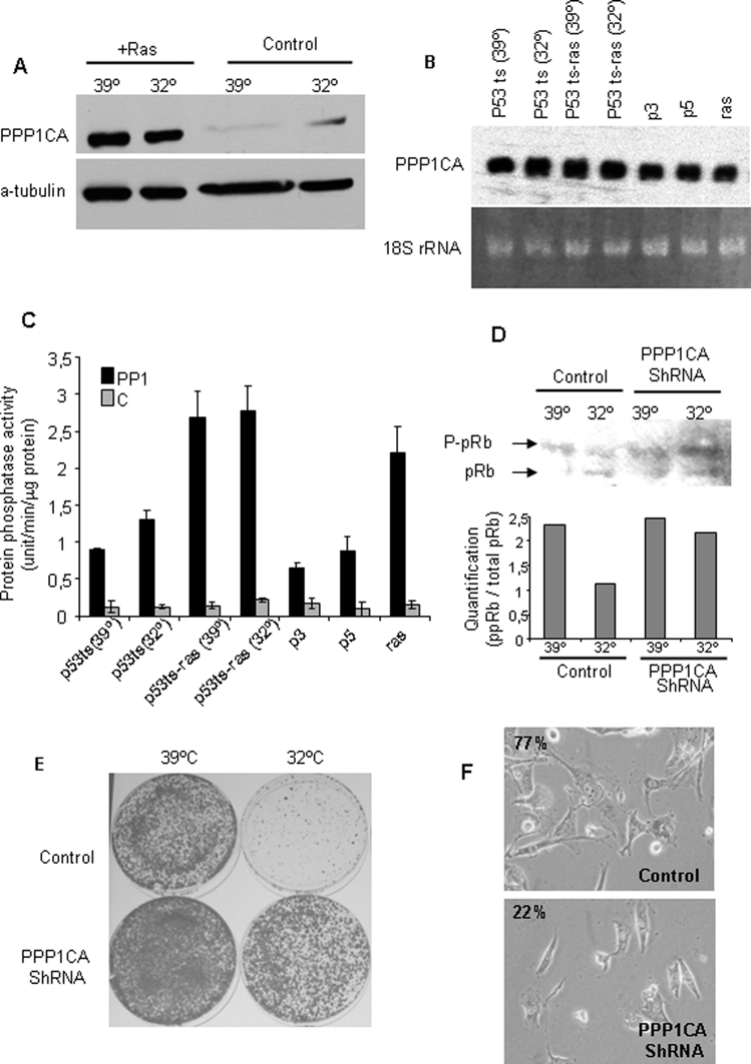
A) Oncogenic Ras increased PPP1CA protein levels. P53ts (control) or p53ts-Ras (+ras) cells were grown at 39°C or incubated at 32°C for 24 hrs. Then PPP1CA protein levels were analyzed by western Blot. α-tubulin was used as a loading control. The data are representative of three independent experiments. B) PPP1CA mRNA levels were not dependent on the expression of oncogenic ras. mRNA levels were analyzed by Northern blot. A labeled probe able to specifically recognize PPP1CA isoform was used as described in M&M. C) Oncogenic ras increased PP1 activity. Exponentially growing cells were keep growing or switch for 24 hrs at 32°C as indicated. Then were starved and PP1 phosphatase activity was measured as described in M&M. C shows the remaining activity after 100 nM okadaic acid treatment to inhibit PP1 and PP2A activity. D, E, and F) P53ts-Ras cells carrying the shRNA against PPP1CA (shRNA) or vector alone (control) were grown at restricted temperature (39°C), or permissive temperature (32°C) as indicated, for 24 hrs. Cells were harvested for protein extraction (for D), fixed and stained with crystal violet (for E) or for SA β-GAL (for F). D) Downregulation of PPP1CA inhibits p53-induced pRb hypophosphorylation. Cells were processed for western blot, showing hyperphosphorylated (ppRb) and hypophosphorylated (pRb) forms of the protein. α-tubulin was used as a loading control. The data are representative of three independent experiments. Bottom panel shows quantification of pRb bands. E) and F) Downregulation of PPP1CA bypasses p53/ras-induced senescence. Cells (10^4^) were seeded and grown at 39° or 32°C for 1 week, then fixed and stained for colony formation with crystal violet (E) or SA β-GAL (F). In F, numbers show the percentage of cells with SA β-GAL staining.

**Figure 6:**
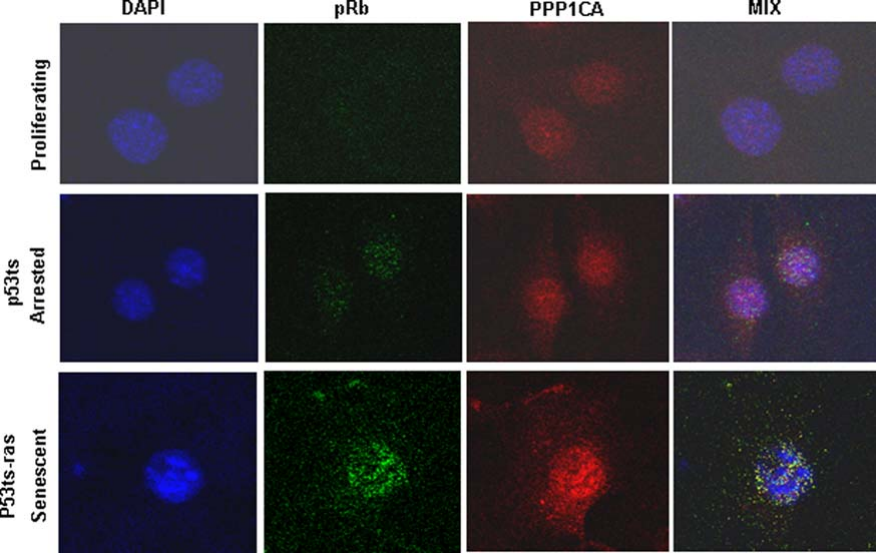
PPP1CA and pRB co-localize during p53/ras-induced senescence. P53ts or p53ts-Ras were grown at 39°C or incubated at 32°C for 24 hrs. Cells were fixed and labeled with DAPI to identify the nuclei, as well as antibodies against PPP1CA (red) or pRb (green).

PPP1CA protein levels increase upon Ras activation (Figure 5A) [34], [18], but not mRNA (Figure 5B). PP1 phosphatase activity also increases upon oncogenic ras expression (Figure 5C), paralleling protein levels of PPP1CA. Expression of a specific shRNA against PPP1CA impairs pRb dephosphorylation, thus bypassing p53-induced arrest. When p53ts-Ras cells were shifted at 32°C, pRb became hypophosphorylated, in accordance with the growth-arrest induced by thermo-sensitive p53 at this permissive temperature. In contrast, p53ts-Ras cells stably transduced with shRNA against PPP1CA showed an increase in the hyperphosphorylated form of Rb protein when kept for 24 h at 32°C (Figure 5D). These data show that downregulation of PPP1CA maintains pRb in the hyperphosphorylated state, even in the presence of active p53, therefore allowing cell growth (Figures 5D and 5E). While p53ts-Ras cells at 32°C show mostly the senescent phenotype, only 22% of cells carrying the PPP1CA shRNA showed senescence features, confirming the relevance of PP1α activity to the senescence phenotype (Figure 5F). This was further confirmed by immunofluorescence studies (Figure 6). In proliferating cells, PPP1CA and pRb levels are low, increasing slightly upon growth arrest. However, these proteins showed diffuse distribution (Figure 6). Under conditions inducing senescence (p53ts-Ras at 32°C), cells increase pRb and PPP1CA levels, which showed nuclear co-localization, strengthening evidence for their functional relationship to senescence.

### Oncogenic stress enhances p53-dependent transcription

**Figure 7:**
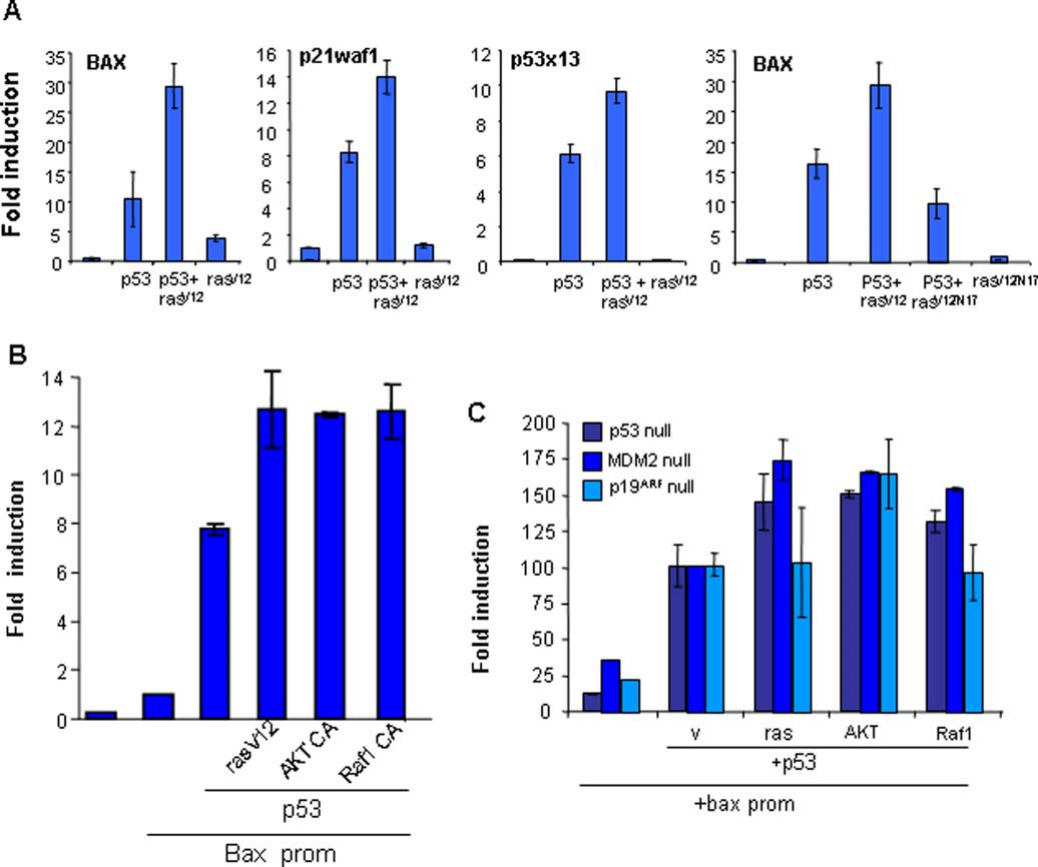
Oncogenic Ras enhances p53-transcriptional activation. A, B and C) p53(−/−), p53(−/−); MDM2(−/−) or p19(−/−) MEFs were transfected as indicated with plasmids carrying luciferase and the indicated genes. Luciferase activity was measured as indicated in the M & M. A) Assays were performed in p53(−/−) MEFs using different promoters responding to p53 (p21waf1, Bax and synthetic p53x13 carrying only the p53 binding site repeated 13 times). B) Only the Bax promoter was used in p53(−/−) MEFs. C) Only the Bax promoter was used in p53(−/−), double p53(−/−); MDM2(−/−) or p19(−/−) MEFs. Data show the averages of three independent experiments.

We also observed that oncogenic Ras enhances p53-dependent transcription (Figures 2A and 2C). To study this effect in detail, we selected three different p53-responsive promoters, Bax, p21waf1 and the synthetic p53 response element (x13). We engineered a construct fusing the different promoters 5′ of the luciferase reporter gene and compared the effect of p53 alone to the effect of the combination of p53 and Ras-val12 (Figure 7A). Oncogenic Ras enhances p53-dependent transcription in all cases, but does not alter transcription when transfected alone (Figure 7A). These effects are dependent on p53 and Ras doses (Figure S2).

To further study this effect, we selected the Bax promoter and investigated dependence of the phenomenon on Ras. To that end, we tested the N17 mutant of Hras-val12. This mutant lacks the ability to bind to Ras effectors and therefore acts as a dominant negative mutant. The N17 mutant does not alter the p53 response (Figure 7A), indicating that the Ras effect is dependent upon activation of Ras effectors. To directly discriminate between the two main effector pathways involved in this effect, the same experiment was performed with active PI3K or Raf pathway mutants. We co-transfected p53 and an active mutant of AKT (AKT-CA) (PI3K pathway), or an active mutant of Raf (BXB-Raf-CAAX). We were able to reproduce the ras-enhancing effect (Figure 7B), indicating that a strong activation of either pathway may provoke the enhancement of p53 transcription.

Ras, acting through the Raf pathway, may activate p53 through p19ARF, either dependent upon or independently of MDM2, while PI3K may inhibit p53 through MDM2 phosphorylation [7], [35], [36]. To determine whether MDM2 or p19 was involved in the effect, the experiment was performed in p19-null or MDM2-null cells (Figure 7C). We observed that the p53-enhancing effect observed in the Ras oncogenic signal was dependent upon p19ARF but not on MDM2. A similar observation was made with the activated Raf oncogene. However, activated AKT showed p53-enhancing effects independent of p19ARF and MDM2 (Figure 7C). These data match those previously reported [11], [37]–[39]; Ras and Raf oncogenes require the p19ARF protein to activate p53.

## Discussion

The data presented in this study elucidate the regulation of p53-responsive genes during proliferation and senescence. We have clearly demonstrated that Ras effects on p53-dependent transcriptional activation result in quantitative rather than qualitative changes. Therefore, the senescence response depends on factors other than p53 activation. p53 activation seems to be necessary but not sufficient to induce senescence, as other signals may be needed for the full onset of senescence. We have shown that Ras-induced activation of PPP1CA, the catalytic subunit of PP1α, is necessary to induce Ras-dependent senescence [18]. It is therefore possible to split the senescence response into two physiological processes. The first of these involves induction of growth arrest and is dependent on p53 activation or other physiological signals activating a proliferative brake similar to that of p53, such as p73 or p63. The second process occurs later, acting on pRb to stabilize its active unphosphorylated form, independent of p53. Unphosphorylated pRb will bind and inactivate E2F factors blocking cell cycle progression and altering local chromatin [27]. PPP1CA activation will take part in this second process, contributing to irreversible proliferative arrest by enforcing pRb dephosphorylation.

Since senescence is a safeguard mechanism that may prevent pretumoral cells from further expansion, many studies have recently emphasized the relevance of this possible new therapeutic tool against cancer (reviewed in [41]–[43]). Our work has identified a set of p53 target genes that affect growth arrest in response to p53 activation. Although our work only identify these 4 genes as the minimal footprint to differentiate growing from p53-arrested cells, these 4 genes have been broadly studied and its relevance in growth arrest and senescence has been established.

Tumor suppressor Lats2 has been shown to be necessary for culture-induced replicative senescence in MEFs, since Lats2−/− MEFs bypass this process [48]. Furthermore, cells lacking Lats2 showed increased prevalence of micronuclei, chromosomal defects and aneuploidy [48], [49]. Lats2 and p53 establish a positive feedback loop that prevents tetraploidization of cells treated with the microtubule poison nocodazole [49]. Most important, miRNA-372 and miRNA373 microRNAs directly target Lats2 expression and have been shown to cooperate with oncogenic Val12-Ras in a way that resembles p53 inactivation, acting as oncogenes in testicular germ cell tumors [50]. Finally, Lats2 has been shown down-regulated through promoter hypermethylation [51], [52], in association with poor prognosis human breast cancers and acute lymphoblastic leukemia. Lats2 might have a role against cancer development, probably through the induction of senescence, and this could explain the link between its down-regulation and tumoral progression. The tumor-suppressor gene RB1 can suppress S phase entry and cause a transient G_1_ arrest following DNA damage [53]–[55] and the mutations in Rb1 pathway-related genes are associated with poor prognosis in many tumor types. The PTEN/PI3K pathway is also regarded as an effector of cellular senescence [56] through p27kip1 cell cycle inhibitor activation.

The key findings obtained in this study may contribute to the current understanding of the molecular basis of senescence and should be of great interest in future senescence studies.

## Materials and Methods

### Cell Culture

Primary MEFs from p53−/− mice were derived from day 13.5 embryos. Cells expressing murine p53val135 were generated by retrovirus-mediated gene transfer of p53val135 into p53−/− MEFs (p53−/− ts). Cells expressing Val12-Ras were generated by retrovirus-mediated gene transfer of pWZLBlast hVal12-Ras into wild-type MEFs at passage 3 (P3-Ras) and p53−/− ts (p53−/− ts Ras cells). Cells were cultured in Dulbecco's Modified Eagle's medium (GIBCO) supplemented with 10% fetal bovine serum (Sigma), 1% penicillin G- streptomycin sulfate (GIBCO), 0.5% fungizone-amphotericin B (GIBCO) and 5 µg/ml plasmocin (InvivoGen).

P53−/− MEFs, p53−/− MDM2−/− MEFs and p19−/− MEFs were cultured in Dulbeccós Modified Eaglés medium (GIBCO). All media was supplemented with 10% fetal bovine serum (Sigma), 1% penicillin G-streptomycin sulphate (GIBCO), and 0.5% fungizone-amphotericin B (GIBCO) in a humidified CO_2_ incubator at 37°C.

### Retroviral Vectors and Gene Transfer

The following retroviral vectors were used: p53val135 mutant cDNA in pWZLHygro and pWZLBlast hVal12-Ras. Retrovirus-mediated gene transfer was performed as previously described [44]. Briefly, 5×10^6^ LinXE retrovirus producer cells were plated in a 10 cm dish, incubated for 24 h and then transfected by calcium-phosphate precipitation with 20 µg of retroviral plasmid (16 h at 37°C). The medium was changed and the plates were maintained at 32°C for 48 h to increase viral stability. Virus-containing supernatant was filtered through a 0.45 µm filter and supplemented with 8 µg/ml polybrene (Sigma) and an equal volume of fresh medium. Prior to infection, 8×10^5^ target fibroblasts were plated per 10 cm dish and incubated for 24 h. For infections, the culture medium was replaced by the viral supernatant, and then the culture plates were centrifuged (1 h at 1,500 rpm) and incubated at 37°C for 16 h. The medium was changed and cells were split 24 h later. Infected cell populations were selected in hygromycin (20 µg/ml) for pWZLHygro-based vectors and in blasticidin (2 µg/ml) for pWZLBlast-based vectors.

### Northern assays

Total RNA was isolated from cells using the TRI-REAGENT method (Molecular Research Center, Cincinnati, OH) according to the manufactureŕs instructions. A reverse transcription was done for each sample (20 µg of total RNA) using MMLV reverse transcriptase (Promega), oligo dT primer and dCTP^32^-labeling nucleotide.

The cDNA ^32^P-labeled probes were hybridized to the p53 target gene array membrane (TranSignal, Panomics, CA, USA) at 42°C overnight. After removing excess substrate by gently washing twice with 2×SSC+0.5% SDS and 0.1×SSC+0.5% SDS at 62°C, the membranes were exposed to BioMax Films (Eastman Kodak Company, NY, USA). The assay normalization was done selecting β-actin as the control housekeeping gene. Analysis was done using the GS-800 Calibrated Densitometer® and the Quantity One® program from Bio-Rad.

Each experiment for each condition was performed independently at least twice, the data quantified and normalized for the value of β-actin (a gene with transcription that is independent of p53).

Raw data for all conditions were normalized against an internal control, β-actin, and then compared to normal proliferating MEFs.

#### PPP1CA Northern Blot

Total RNA was extracted using RNAzolB. 10 µg of total RNA were run in formaldehyde-agarose gels and transferred to a Hybond membranes. The membrane was pre-hybridized during 4 hours at 65°C. The probe was labeled by PCR with 50 µC of redivue dCTP32 (Amersham), using specific primers for mouse PPP1CA. The purified probe was denatured and added to the hybridization solution. The hybridization was performed overnight at 65°C. After extensive whashing, the membrane was exposed to a Biomax MS film (Kodak).

### Data Analysis

The data consisted of the expression values of 122 transcriptional targets of p53 in different cellular conditions, which led either to proliferation or to growth arrest.

#### Clustering analysis

Hierarchical clustering was performed using the function hcluster (package amap) of the free statistical software R (Ihaka and Gentleman, 1996). Before statistical analysis, gene expression levels were standardized gene by gene across all conditions using the median and interquartile range (IQR). The cellular conditions were clustered using Ward linkage and uncentered Pearson metric tests. The results were visualized and analyzed with TreeView (M. Eisen; http://www.microarrays.org/software). The expression level of each gene, relative to its median expression level across all conditions, was represented by a color, with red representing expression greater than the median, green representing expression less than the median, and color intensity representing the magnitude of the deviation from the median.

#### Feature selection

The problem of extracting a robust set of predictors for the proliferating status of the different cellular conditions has been formulated as a least squares regression problem. Since the number of genes is much larger than the number of conditions, we used penalized regression methods. The standard penalty used in so-called ridge regression is given by the L2-norm of the vector containing the regression coefficients. Such penalty allows stabilizing the ordinary least squares estimate, but typically will retain all regression coefficients so that no selection of the relevant variables (genes) may be done. To perform the selection task, we used an L1-norm penalty, as is done in lasso regression. This type of penalty is known indeed to promote sparsity, i.e., to force many regression coefficients to be zero; this obviates the need for pre-selection of the data. However, a known drawback of the L1 penalty for variable selection is that in a group of highly correlated genes, it may pick up only one representative. We therefore also used combined L1- and L2-norm penalties to select sparse groups of highly correlated genes; this is done in the so-called “elastic net” proposed in Zou and Hastie, 2005, [46]. To compute the corresponding penalized least-squares solutions, we applied the iterative thresholding algorithm developed in Daubechies et al, 2004. [47], which is simple to implement, robust to measurement errors and works well for high-dimensional data. Despite the small number of conditions, some standard validation tests (such as leave-one-out, label and gene permutation, bootstrap sampling) were performed.

### Transcriptional Assays

For transient transfection of cells, we seeded 2–4×105 H1299 cells per well in six-well plates. After 24 h, transfections were performed by the calcium chloride method and JetPEI reagent (Polytransfection, Illkirch, France) according to the manufacturer's recommendations. For both transfection methods, we used 1.5–2 µg each of the reporter plasmids pGL3-13X, pGL3-Bax and pGL3-p21 in the presence or absence of pBABE puro p53 wt (0.6–0.75 µg) and pLSXN Ras val 12, or active mutants of the PI3K or Raf pathway (0.6–0.75 µg).

Renilla luciferase plasmid was used as an internal control for transfection efficiency. The total amount of DNA within the experiments was kept constant by adding empty vector plasmid DNA to the transfection mixtures.

Reporter gene assays were performed with the Dual-Luciferase® Reporter Assay System (Promega, USA) 48 h after transfection and the results were measured with a Victor2V luminometer. The activity of the reporter luciferase was expressed relative to the activity in renilla vector-transfected cells. Similar results were obtained in at least three different experiments. All results were compared to the control and are shown in the figures as the mean±S.D. of independent triplicate cultures.

### Western Blot

Cells were prepared in lysis-buffer and proteins were separated on SDS-PAGE gels, transferred onto PVDF membranes (Immobilon-P, Millipore) and immunostained. The following primary antibody was used: anti-p53FL393 (Santa Cruz 6243, diluted 1∶1000), anti-PP1α (from Calbiochem) anti-Rb: G3-245 from BD Pharmingen; and horseradish peroxidase-labeled rabbit anti-mouse (Promega diluted 1∶5000) and goat anti-rabbit (Calbiochem 401315, diluted 1∶4000) secondary antibodies. Proteins were visualized using the ECL detection system (Amersham Biosciences, Buckinghamshire, UK).

### Immunofluorescence

#### Immunostaining and confocal analysis for 53BP1 and γH2AX foci

Cells were seeded onto glass cover slips and cultured for 8 h at 39°C. Then we placed the cells at 39°C and 32°C. After 24 h (cells at 39°C and 32°C) and 48 h, 96 h and 144 h (cells at 32°C), cover slips were fixed in 4% paraformaldehyde for 5 min at room temperature, washed twice with PBS, permeabilized in Triton X-100 0.5% in PBS for 5 min and washed twice more with PBS. Samples were incubated in blocking solution (PBS containing 3% bovine serum albumin) at 37°C for 15 min, followed by incubation for 30 min at 37°C with anti-phospho-Histone H2A.X (Ser139) antibody (Millipore 05-636) or anti-53BP1 antibody (Novus Biologicals NB100-304) diluted 1∶100. After washing with PBS, cells were incubated with species-specific Alexa 488-conjugated secondary antibody diluted 1∶100 in blocking buffer for 30 min at 37°C in the dark. The nuclei were stained with Hoechst 33258 diluted 1∶1000 for 3 min at room temperature in the dark prior to mounting with mowiol (Calbiochem). Images were collected by confocal laser microscopy (model TCS-SP2-AOBS, Leica, Germany).

#### Immunostaining and confocal analysis for PPP1CA and pRb co-localization

Cells were seeded onto glass cover slips and cultured for 8 h at 39°C. Then we placed the cells at 39°C and 32°C. After 24 h (cells at 39°C and 32°C) and 48 h (cells at 32°C), cover slips were fixed in 4% paraformaldehyde for 5 min at room temperature, washed 2 times with PBS, permeabilized in Triton X-100 0.5% in PBS for 5 min and washed again 2 times with PBS. Samples were incubated in blocking solution (PBS containing 3% bovine serum albumin) at 37°C for 15 min, followed by incubation for 30 min at 37°C with anti-human Retinoblastoma Protein (RB) monoclonal antibody (BD Pharmingen 554136) diluted 1∶100. After washing with PBS, cells were incubated with species-specific Alexa 488-conjugated secondary antibody diluted 1∶100 in blocking buffer for 30 min at 37°C in the dark. Then, cells were incubated with anti-Protein Phosphatase 1α, C-terminal antibody (Calbiochem 539517) diluted 1∶100. After washing with PBS, cells were incubated with species-specific Alexa 633-conjugated secondary antibody diluted 1∶100 in blocking buffer for 30 min at 37°C in the dark. The nuclei were stained with Hoechst 33258 diluted 1∶1000 for 3 min at room temperature in the dark prior to mounting with mowiol (Calbiochem). Images were collected by confocal laser microscopy (model TCS-SP2-AOBS, Leica, Germany).

### SA ß-Gal activity

Senescence-associated (SA) ß-galactosidase (ß-Gal) activity was measured as previously described [45], except that cells were incubated in 5-bromo-4-chloro-3-indolyl-ß-D-galactopyranoside (XGal) at pH 5.5 to increase the sensitivity of the assay in MEFs. The percentage of cells expressing SA ß-Gal was quantified by inspecting >400 cells per 10-cm-diameter plate three times.

### Protein phosphatase assays

PP1 activity was determined according to standard procedures as previously described [57]. PP activity was assayed using 32P-labeled phosphorylase a as a substrate which detects both PP1 and PP2A activities. To selectively measure PP1 activity we used 2 nM okadaic acid to selectively inhibit PP2A. The cell pellet was homogenized in the extraction buffer containing 20 mM Tris-HCI, pH 7.5, 5 mM EDTA, 10 mM EGTA, 15 mM -mercaptoethanol, 0.25 M sucrose, 0.3% Triton X-100, 5 µg/ml leupeptin, and 5 µg/ml aprotinin and centrifuged to give a soluble supernatant. The PP activity in the clear supernatant was determined by measuring the trichloroacetic acid-soluble counts released after incubation of the 32P-labeled phosphorylase a in the cell extract. The PP activity was linear up to assay times of 10 min and 5 µg protein of the cell extract. Routinely, incubation for PP activity was carried out for 10 min with an extract containing 5 µg of protein as determined by the Bio-Rad assay (Bio-Rad, Hercules, CA). Negative controls were obtained incubating with 100 nM Okadaic acid to inhibit PP1 and PP2A activity. One unit (U) of activity is defined as the amount that catalyzes the release of 1 nmol Pi from phosphorylase a per min at 30°C.

### Real time PCR (qRT-PCR) experiments

Total RNA were isolated form HCT 116 p53 +/+ cells (a generous gift from B. Vogelstein) treated with 400nM and 1 microM Etoposide, 0.6 µg/ml Doxorubicin, 10nM Paclitaxel (Taxol), 100nM UCN-01, 15 µM PD98059 for 8 hours. After DNAse treatment, reverse transcription was performed with 20 µg of mRNA using MMLV reverse transcriptase (Promega) and oligo dT primer according to the manufacturer's recommendations

QRT- PCR experiments were carried out using SYBR® Green PCR Master Mix (Applied Biosystems, USA). Reaction mixtures contained: 5 µl cDNA sample (1/10 dilution RT product), 1.5 µl primer mix (sense and antisense, 0.6 µM final concentration), and 12.5 µl SYBR® Green PCR Master Mix. The final volume should be 25 µl. The following primers were used to amplify regions: LATS2 forward 5′- AACAGCCTCAACGTGGACCTGTATGAA-3′ and reverse 5′-CAGGGCATGCTCCTCCTTGGCGTCGAA- 3′; PTEN forward 5′-CAGAAAGACTTGAAGGCGTAT-3′ and reverse 5′- GTAACGGCTGAGGGAACT C-3′; RB1 forward 5′-TCTGCATTGGTGCTAAAAGTTTCTTGGA-3′ and reverse 5′-CCTGTTCTGACCTCGCCTGGGTGTTCGA- 3′; MAP4 forward 5′-TGATCCCTTTAAGATGTACCATGATGAT-3′and reverse 5′-AATGCTTGTGCTGGTGGCCTCTCTTCTG-3′ and β-actine forward 5 -AGGCCAACCGCGAGAAGATGAC-3 and reverse 5 -GAAGTCCAGGGCGACGTAGCA-3′. The samples were amplified according to the following protocol: 10 min 95°C, 50 cycles: 15 sec 95°C, 30 sec 56°C–62°C (depending on the primer), 1 min 72°C. Then in order to get the dissociation curve, a stage was added: 15 sec 95°C, 15 sec 60°C and 15 sec 95°C.

The normalized values were analyzed using SDS2.2.2 program (Applied Biosystems, USA). All samples were measured in duplicates and the right formation of the products was verified by 1% agarose gel electrophoresis (data not shown).

## Supporting Information

Figure S1 List of 122 p53 target genes used int his study
(0.11 MB EMF)

Figure S2 Oncogenic ras increases p53-induced transcription in a dose-dependent maner
(0.07 MB TIF)
