# Supplementary figures and images for: Characterization of the p53 Response to Oncogene-Induced Senescence
Source: PLoS One. 2008 Sep 18;3(9):e3230. doi: 10.1371/journal.pone.0003230 (PMC2535567; doi:10.1371/journal.pone.0003230)

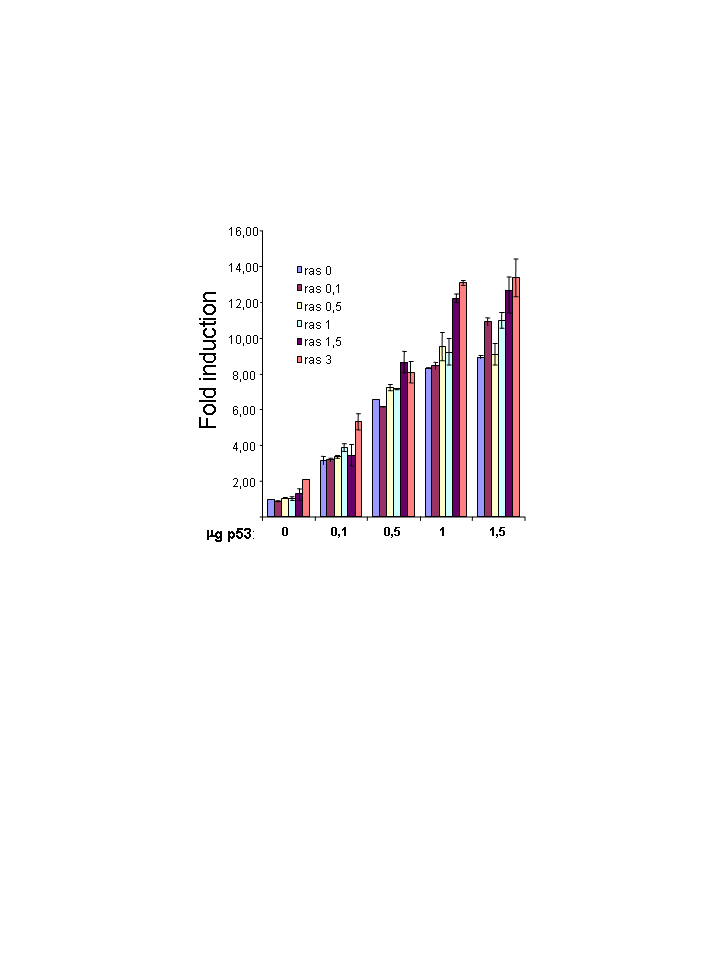

Supplement: Figure S2 — Oncogenic ras increases p53-induced transcription in a dose-dependent maner (0.07 MB TIF) [file pone.0003230.s002.tif]
